# Supplementary material for: The Systems Biology Research Tool: evolvable open-source software
Source: BMC Syst Biol. 2008 Jun 29;2:55. doi: 10.1186/1752-0509-2-55 (PMC2446383; doi:10.1186/1752-0509-2-55)
Supplement: Additional file 1 — SBRT Archive. An archive of the current version of the Systems Biology Research Tool. [file 1752-0509-2-55-S1.zip › sbrt-1.4.0/doc/developers_guide/api/sbrt/kernel/math/algebra/class-use/SparseDoubleMatrix.html]

Uses of Interface sbrt.kernel.math.algebra.SparseDoubleMatrix


|  |  |  |
| --- | --- | --- |
| **Overview** \| **Package** \| **Class** \| **Use** \| **Tree** \| **Deprecated** \| **Index** \| **Help** | |  |
| PREV   NEXT | **FRAMES**    **NO FRAMES**     **All Classes** |  |


---


## **Uses of Interface sbrt.kernel.math.algebra.SparseDoubleMatrix**

| Packages that use SparseDoubleMatrix | |
| --- | --- |
| **sbrt.kernel.fba** | Provides classes and interfaces for creating objects relevant to Flux Balance Analysis. |
| **sbrt.kernel.fba.comp** | Provides classes and interfaces for performing Flux Balance Analysis computations. |
| **sbrt.kernel.fba.proc** | Provides classes and interfaces for executing Flux Balance Analysis processes. |
| **sbrt.kernel.math.algebra** | Provides classes and interfaces for creating objects relevant to algebra. |
| **sbrt.shell.io** | Provides classes and interfaces for reading and writing files. |

Uses of SparseDoubleMatrix in sbrt.kernel.fba

| Methods in sbrt.kernel.fba that return SparseDoubleMatrix | |
| --- | --- |
| `SparseDoubleMatrix<java.lang.String,java.lang.String>` | `Fluxome.getStoichiometryMatrix()`             Returns the stoichiometry matrix of this fluxome. |
| `SparseDoubleMatrix<java.lang.String,java.lang.String>` | `IrrevRxnFluxomeV1.getStoichiometryMatrix()`             Returns the stoichiometry matrix of this fluxome. |

Uses of SparseDoubleMatrix in sbrt.kernel.fba.comp

| Methods in sbrt.kernel.fba.comp that return SparseDoubleMatrix | |
| --- | --- |
| `SparseDoubleMatrix<?,java.lang.String>` | `SlpCycleComputer.getCycles(IrrevRxnFluxome fluxome)`             Returns the set of all steady state cycles (Type III extreme pathways) contained in the provided fluxome. |
| `SparseDoubleMatrix<?,java.lang.String>` | `CycleComputer.getCycles(IrrevRxnFluxome fluxome)`             Returns the set of all steady state cycles contained in the provided fluxome. |
| `SparseDoubleMatrix<?,java.lang.String>` | `SlpExCurrComputerV2.getMatrix(IrrevRxnFluxome fluxome)`             Returns the extreme current matrix for the provided fluxome. |
| `SparseDoubleMatrix<?,java.lang.String>` | `ExtremeCurrentComputer.getMatrix(IrrevRxnFluxome fluxome)`             Returns the extreme current matrix for the provided fluxome. |
| `SparseDoubleMatrix<?,java.lang.String>` | `SlpExCurrComputerV2.getMatrix(IrrevRxnFluxome fluxome, MutableProgressMonitor pm)`             Returns the extreme current matrix for the provided fluxome. |
| `SparseDoubleMatrix<?,java.lang.String>` | `ExtremeCurrentComputer.getMatrix(IrrevRxnFluxome fluxome, MutableProgressMonitor pm)`             Returns the extreme current matrix for the provided fluxome. |

Uses of SparseDoubleMatrix in sbrt.kernel.fba.proc

| Methods in sbrt.kernel.fba.proc that return SparseDoubleMatrix | |
| --- | --- |
| `SparseDoubleMatrix<java.lang.String,java.lang.String>` | `NetworkInfoProcess.Results.getStoichMatrix()`             Returns the stoichiometry matrix of the fluxome. |

Uses of SparseDoubleMatrix in sbrt.kernel.math.algebra

| Subinterfaces of SparseDoubleMatrix in sbrt.kernel.math.algebra | |
| --- | --- |
| `interface` | `SmdMatrix<R,C>`             This interface is used to represent sparse mutable matrices containing strictly double precision numbers as elements. |

| Classes in sbrt.kernel.math.algebra that implement SparseDoubleMatrix | |
| --- | --- |
| `class` | `AbstractSmdMatrix<R,C>`             This abstract class is a skeleton implementation of `SmdMatrix`. |
| `class` | `HashMatrix<R,C>`             This class is a `java.util.LinkedHashMap` based implementation of `SmdMatrix`. |
| `class` | `RowOrTreeMatrix<R extends Comparable<? super R>,C extends Comparable<? super C>>`             This class is a `java.util.TreeMap` based implementation of `SmdMatrix`. |
| `class` | `SparseDoubleMatrixWrap<R,C>`             This class is used to wrap mutable matrices as an immutable `SparseDoubleMatrix`. |
| `class` | `TreeMatrix<R extends Comparable<? super R>,C extends Comparable<? super C>>`             This class is a `java.util.TreeMap` based implementation of `SmdMatrix`. |

| Methods in sbrt.kernel.math.algebra that return SparseDoubleMatrix | |
| --- | --- |
| `SparseDoubleMatrix<R,C>` | `SparseDoubleMatrix.getColumn(java.lang.Object columnIndex)`             Returns the column corresponding to the provided column index. |
| `SparseDoubleMatrix<R,C>` | `SparseDoubleMatrixWrap.getColumn(java.lang.Object columnIndex)`             Returns the column corresponding to the provided column index. |
| `SparseDoubleMatrix<R,C>` | `SparseDoubleMatrix.getRow(java.lang.Object rowIndex)`             Returns the row corresponding to the provided row index. |
| `SparseDoubleMatrix<R,C>` | `SparseDoubleMatrixWrap.getRow(java.lang.Object rowIndex)`             Returns the row corresponding to the provided row index. |

| Methods in sbrt.kernel.math.algebra with parameters of type SparseDoubleMatrix | |
| --- | --- |
| `<R,C,T> void` | `MatrixMultiplier.multiply(SparseDoubleMatrix<R,C> matrixA, SparseDoubleMatrix<C,T> matrixB, DoubleMatrixBuilder<R,T> product)`             Fills `product` with `matrixA` \* `matrixB`. |
| `<R,C,T> void` | `MatrixMultiplier.multiply(SparseDoubleMatrix<R,C> matrixA, SparseDoubleMatrix<C,T> matrixB, DoubleMatrixBuilder<R,T> product)`             Fills `product` with `matrixA` \* `matrixB`. |
| `void` | `SmdMatrix.setElements(SparseDoubleMatrix<? extends R,? extends C> m)`             Sets the corresponding elements of this matrix to those of the provided matrix. |
| `void` | `AbstractSmdMatrix.setElements(SparseDoubleMatrix<? extends R,? extends C> m)`             Sets the corresponding elements of this matrix to those of the provided matrix. |

Constructors in sbrt.kernel.math.algebra with parameters of type SparseDoubleMatrix

`SparseDoubleMatrixWrap(SparseDoubleMatrix<R,C> m)`
  
          Constructs a new wrapper around the provided matrix.

Uses of SparseDoubleMatrix in sbrt.shell.io

| Methods in sbrt.shell.io that return SparseDoubleMatrix | |
| --- | --- |
| `static SparseDoubleMatrix<java.lang.Integer,java.lang.String>` | `ComiFile.getMatrix(ComiFile<java.lang.String,java.lang.Double> file)`             Reads the provided column-oriented map input file and returns its contents as a sparse matrix. |

---


|  |  |  |
| --- | --- | --- |
| **Overview** \| **Package** \| **Class** \| **Use** \| **Tree** \| **Deprecated** \| **Index** \| **Help** | |  |
| PREV   NEXT | **FRAMES**    **NO FRAMES**     **All Classes** |  |


---
